# Complete Genome Sequence of Porcine Deltacoronavirus Strain CH/Sichuan/S27/2012 from Mainland China

**DOI:** 10.1128/genomeA.00945-15

**Published:** 2015-09-03

**Authors:** Yi-Wen Wang, Hua Yue, Weihuan Fang, Yao-Wei Huang

**Affiliations:** a Institute of Preventive Veterinary Medicine, College of Animal Sciences, Zhejiang University, Hangzhou, Zhejiang Province, China; b College of Life Science and Technology, Southwest University for Nationalities, Chengdu, Sichuan Province, China

## Abstract

We report the first complete genome sequence of porcine deltacoronavirus (PDCoV) strain CH/Sichuan/S27/2012 identified in feces of diarrheic piglets from mainland China in 2012. This strain has two unique in-frame deletions within the ORF1a gene and is phylogenetically between the prototype PDCoV (HKU15) and the 2014 U.S. strains.

## GENOME ANNOUNCEMENT

Porcine deltacoronavirus (PDCoV) is a positive-sense single-stranded RNA virus belonging to the proposed fourth genus, *Deltacoronavirus*, in the family *Coronaviridae* (1). The first two genomic sequences of PDCoVs, designated HKU15-44 and HKU15-155, were first identified in swine from Hong Kong in the period 2007 to 2011 (2). In early 2014, the Ohio Department of Agriculture reported the presence of a PDCoV in the United States. The full-length genomic sequence of this strain, named OH1987, was closely related to two HKU15 strains (3). Experimental infection studies confirmed that the U.S. PDCoV strains are enteropathogenic and induced clinical diarrhea in gnotobiotic or neonatal piglets (4). It has been hypothesized that the U.S. porcine epidemic diarrhea virus (PEDV), responsible for a widespread outbreak of PED in the United States since May 2013, might originate from China (5). Although the prototype PDCoV was first identified in Hong Kong, the existence of PDCoV after 2011 in mainland China and its genetic relationship with the U.S. PDCoVs (if one exists) have not been investigated.

Previously, using a metagenomics approach, two (S26 and S27) of twenty-seven fecal samples from diarrheic piglets, collected in the Sichuan province of west China during the second half of 2012, were detected as non-PEDV “coronavirus” positive (6). Further analysis of these two “coronavirus” sequences (471 bp) indicated that they were mapped to the partial ORF1a/1b gene of PDCoV. The complete genomic sequence of PDCoV (CH/Sichuan/S27/2012) from the sample “S27” was subsequently determined from extracted RNA by reverse transcription (RT)-PCR amplification of 16 regions covering the PDCoV genome as described previously (3). The RT-PCR products were individually gel purified and subsequently cloned into a pCR-Blunt vector (Life Technologies, Carlsbad, CA). For each amplicon, five individual clones were sequenced to determine the consensus sequence. The sequences were assembled and analyzed using the Lasergene 11 Core Suite.

The complete genome of the CH/Sichuan/S27/2012 strain is 25,404 nucleotides (nt) in length, excluding the poly(A) tail. The genome organization is similar to those of other reported PDCoVs, with the typical gene order 5′-ORF1a/1b-S-E-M-NS6-N-3′. However, this strain has two unique in-frame deletions within the ORF1a gene that are not shown in the other PDCoVs: a 6-nt (TTTGAA) deletion at positions 1,739 to 1,744 (corresponding to the HKU15-44 sequence) in the nonstructural protein (nsp) 2 region and a 9-nt (GCCGGTTGG) deletion at positions 2,810 to 2,818 in the nsp 3 region. The S27 shared 98.8% and 99.1% nt sequence identities with HKU15-44 and HKU15-155, respectively, and exhibited 98.9 to 99.0% nt identities with the available U.S. PDCoVs. In accordance with this, the S27 sample is phylogenetically between HKU15 and the U.S. strains at the levels of the complete genome, the structural protein region (S-E-M-NS6-N), and the S gene, respectively.

To our knowledge, this is the first complete genome sequence of PDCoV from mainland China since 2012. The sequence data of CH/Sichuan/S27/2012 fill the gap of PDCoV sequences between 2012 and 2013, right before the suspected introduction of PDCoV into North America, which should facilitate further studies on PDCoV evolution.

### Nucleotide sequence accession number.

The complete genome sequence of the PDCoV strain CH/Sichuan/S27/2012 has been deposited in GenBank under the accession number KT266822.
